# TSH suppression therapy from an individualized perspective: from mechanism to clinical decision-making

**DOI:** 10.3389/fendo.2026.1842060

**Published:** 2026-07-07

**Authors:** Zhaoqing Li, Tengfei Wang, Jia Liu

**Affiliations:** 1 Department of Thyroid Surgery, General Surgery Center, The First Hospital of Jilin University, Changchun, China; 2 Department of Hepatobiliary and Pancreatic Surgery, General Surgery Center, The First Hospital of Jilin University, Changchun, China

**Keywords:** clinical decision-making, differentiated thyroid cancer, individualization, postoperative management, TSH suppression therapy

## Abstract

Thyroid-Stimulating Hormone (TSH) suppression therapy is a key strategy in managing differentiated thyroid cancer (DTC) after surgery, but its clinical use has long faced the challenge of a “one-size-fits-all” approach. Previous studies mainly focus on balancing risks and benefits, yet the process of individualized decision-making still lacks systematic integration and synthesis. This article offers a comprehensive review of this issue from an individualized perspective. Mechanistically, it details the networked regulatory mechanisms by which TSH promotes the proliferation, invasion, and dedifferentiation of thyroid cancer cells through the activation of signaling pathways like Gαs and Gαq/11 via TSHR. It also uncovers the complex relationship where TSH collaborates with the key oncogenic drivers to advance tumor growth and the loss of oncogene-induced senescence after long-term stimulation. Clinically, we develop an individualized decision-making framework based on three dimensions: patient factors, tumor traits, and treatment process. Additionally, we synthesize existing evidence to define the benefit boundaries of TSH suppression, aiming to guide clinical practice.

## 1 Introduction

Thyroid cancer, as one of the most common endocrine tumors worldwide, is also the most common cancer among adolescents and adults. Its incidence has been steadily increasing over the past few decades (1). Research suggests that the rise in thyroid cancer incidence is largely attributable to early screening and advances in imaging technology, which have improved the detection and diagnosis of small, indolent tumors that would not cause symptoms or require treatment (2). However, this increased detection rate has also led to overtreatment, complicating standard diagnostic approaches and highlighting the need for personalized treatment plans. Most thyroid cancers originate from follicular epithelial cells of the thyroid and exhibit significant histological heterogeneity and diverse clinical behaviors, ranging from the common indolent classic papillary carcinoma to the rare invasive undifferentiated/anaplastic carcinoma (3). Among these, follicular-derived differentiated thyroid cancers (such as papillary and follicular carcinomas) are the most common types, with a good clinical prognosis and very low mortality (1). Notably, although undifferentiated thyroid carcinoma accounts for only 1.3% of all thyroid cancers, it accounts for 19.9% of thyroid cancer-related deaths, with a median overall survival of 6.5 months (4, 5), which highlights the urgent need for neoadjuvant therapy, targeted therapy, and immunotherapy to improve survival outcomes for these patients (6, 7).

Currently, surgical treatment plays a crucial role in the management of DTC. Meanwhile, postoperative interventions such as radioactive iodine remnant ablation, which is now used mainly for selected intermediate to high-risk patients, and TSH suppression therapy, with its intensity adjusted according to recurrence risk and treatment response, have become important ways to reduce recurrence risk (8). In particular, TSH suppression therapy, as the main approach for postoperative management of DTC, reduces the risk of recurrence by exogenously administering thyroid hormones to achieve normal thyroid function while suppressing TSH through the hypothalamic-pituitary feedback mechanism (9). In the latest 2025 American Thyroid Association (ATA) guidelines, it is proposed that the risk of recurrence in differentiated thyroid cancer should be more accurately assessed based on tumor histopathological characteristics, the number of cervical lymph node metastases, the American Joint Committee on Cancer (AJCC) staging system, postoperative imaging findings, and serum Tg and TgAb results. Furthermore, the degree and duration of TSH suppression should be individualized according to the potential benefits and risks (8).

However, it is worth noting that TSH suppression therapy is not suitable for all patients, and there are significant differences in patients’ responses and tolerance to the treatment (10, 11). Therefore, the concept of individualized medicine offers a new direction for optimizing TSH suppression therapy. Nevertheless, the implementation of individualized TSH suppression therapy faces numerous challenges, including patients’ physiological characteristics, tumor biological features, and sensitivity to treatment side effects. Additionally, differences in patients’ needs regarding treatment goals and quality of life further increase the complexity of clinical decision-making, making the formulation of individualized TSH suppression strategies in clinical practice increasingly important.

This review aims to comprehensively elucidate the mechanisms by which TSH contributes to the initiation and progression of thyroid cancer, and on this basis, to explore in depth the multidimensional factors influencing the individualized implementation of TSH suppression therapy, including patient characteristics, tumor pathology, and treatment-related variables. By integrating and analyzing existing evidence, we hope to provide a more comprehensive decision-making perspective for clinical practice, helping physicians to more accurately identify key influencing factors and define the boundaries of benefit when developing individualized TSH suppression strategies. In doing so, we aim to effectively reduce the risk of recurrence while promoting the transition of postoperative management for differentiated thyroid cancer from empirical treatment to individualized precision medicine.

## 2 The pro-tumorigenic role of TSH in thyroid cancer

**Figure 1:**
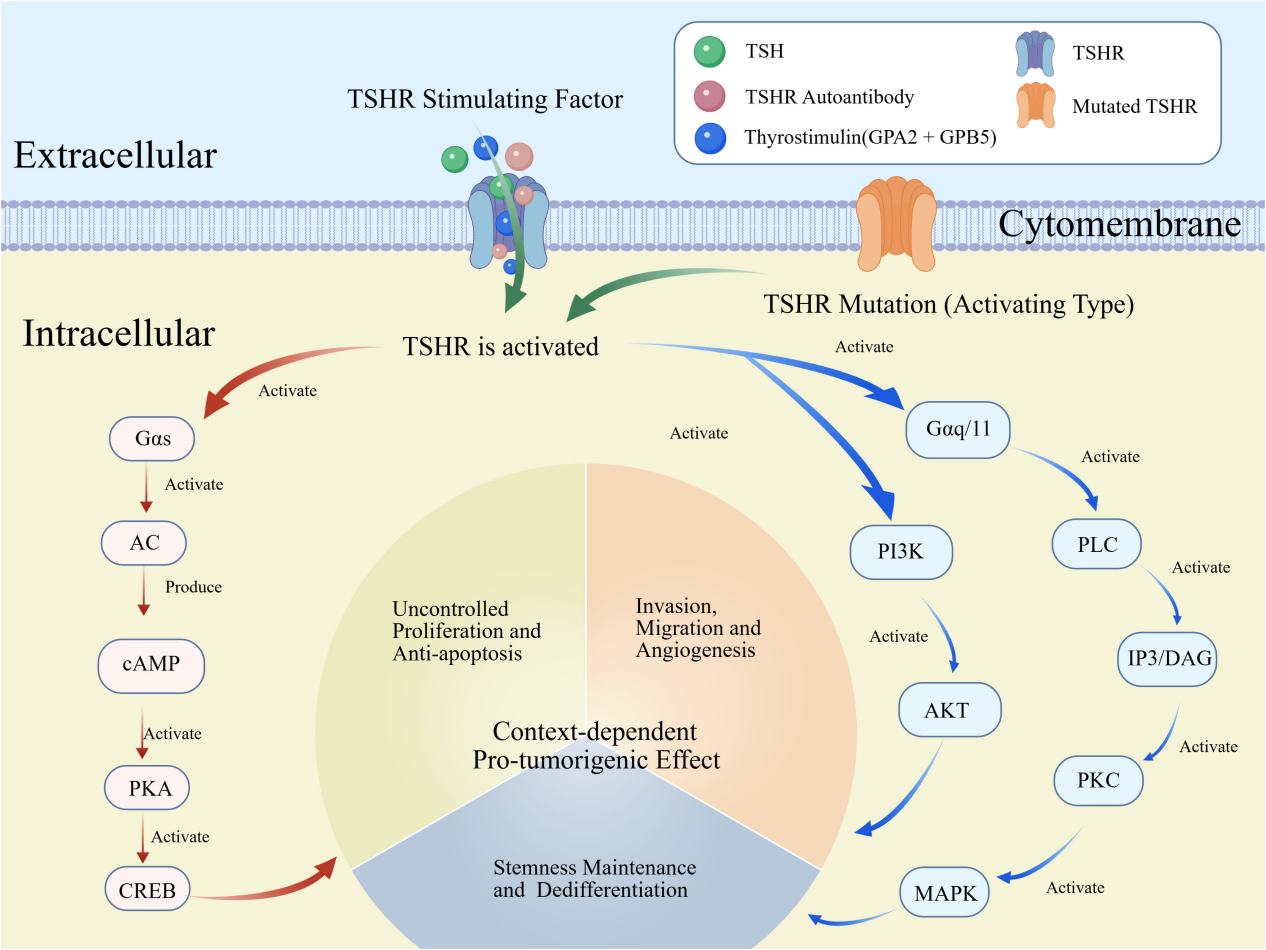
Context-dependent pro-tumorigenic effects of TSH/TSHR signaling in thyroid cancer: TSH/TSHR signals through Gαs and Gαq/11. The effects are context-dependent, determined by TSHR expression levels, tumor differentiation, G protein coupling, and interactions with other oncogenic alterations.

Accumulating experimental studies and clinical data indicate that TSH exerts a significant pro-tumorigenic role in thyroid cancer, with existing evidence more strongly supporting its involvement in disease progression, thereby providing a theoretical basis for TSH suppression as an important measure in the postoperative management of differentiated thyroid cancer (12). As early as 1976, Yoichi Ichikawa and colleagues, using a radioreceptor assay technique, identified the presence of TSH receptors (TSHR) in differentiated thyroid cancer and proposed that the therapeutic responsiveness of thyroid hormone therapy for thyroid cancer could be determined by the presence or absence of TSHR (13). By activating TSHR, TSH can promote proliferation and survival in differentiated, TSHR−expressing thyroid cancer cells. However, TSHR downregulation is associated with a poorer prognosis (14). Deficient TSHR expression promotes epithelial−mesenchymal transition, thereby facilitating invasion and metastasis, and independently predicts distant metastasis and shorter survival (15). Thus, while TSH−TSHR signaling may support tumor growth in TSHR−retaining cells, loss of TSHR marks a more aggressive, less TSH−responsive phenotype. In this process, TSH does not act in isolation, it interacts with other factors, further amplifying its pro-tumorigenic effects. Examples include DNA damage from ionizing radiation, a well−established environmental risk (16). Major molecular drivers in thyroid follicular cell−derived carcinomas include rearrangements such as RET/PTC and PAX8/PPARG, as well as mutations in BRAF, RAS, TERT promoter, TP53, PIK3CA, and AKT1 (17, 18). Hashimoto’s thyroiditis acts as a disease modifier, playing a dual−edged role (19). TSH itself appears to work more as a disease modifier than as a direct trigger of thyroid cancer. These interactions not only reveal the complex role of TSH in the pathogenesis of thyroid cancer but also highlight the importance of multifactorial regulation, providing a more comprehensive perspective for clinical intervention (**Figure 1**).

It is important to recognize that the effects of TSH-TSHR signaling in thyroid cancer are context-dependent, depending on TSHR expression levels, tumor differentiation, G-protein coupling, and interactions with other oncogenic alterations. In differentiated thyroid cancer, canonical TSHR-Gαs signaling supports thyroid-specific functions such as iodine uptake and hormone synthesis, and can also contribute to regulated proliferative responses. However, context-dependent or noncanonical TSH-TSHR signaling—such as Gα12/13-RhoA activation or redirection of Gs-AC-PKA signaling toward JNK-c-JUN and PD-L1 regulation—has been associated with migration, invasion, dedifferentiation, and immune evasion in certain settings (20, 21). These findings indicate that TSH signaling may exert both differentiation-supporting and pro-tumorigenic effects depending on cellular context. At the same time, TSHR expression is often reduced in advanced, poorly differentiated, or radioiodine-refractory tumors and correlates with more aggressive behavior. Experimental evidence also suggests that loss of functional TSHR can enhance invasion and epithelial-mesenchymal transition (15). This duality helps explain why TSH suppression remains clinically relevant (to limit stimulation of TSHR-positive cells), while TSHR expression itself reflects differentiation status and is being explored as a therapeutic target (22). As a G protein-coupled receptor (GPCR), TSHR activates signaling pathways by switching between different G protein families (primarily Gαs and Gαq/11), regulating thyroid cell growth, differentiation, and hormone secretion (23). However, this mechanism can be hijacked in thyroid cancer, leading to pro-tumorigenic effects. Its typical stimulation originates from circulating TSH or autoantibodies (such as stimulating antibodies in Graves’ disease), and different TSHR autoantibodies possess unique signaling imprints, resulting in variable signaling responses and consequently different clinical manifestations of autoimmune thyroid disease (24). Atypical stimulation includes autonomous activation of TSHR caused by TSHR gene mutations, leading to hereditary hyperthyroidism with thyroid hyperplasia in the absence of thyroid autoantibodies or clinical signs of autoimmunity (25). Another atypical stimulator is thyrostimulin (a heterodimeric glycoprotein composed of GPA2 and GPB5), first discovered in 2002. Due to its dual binding with TSHR, it has a higher affinity for TSHR than TSH. At the same time, as an important signaling molecule, it participates in processes such as thyroid stimulation, skeletal development, hormonal system regulation, pathology including ovarian cancer and inflammation, as well as reproductive process regulation (26). These stimulators collectively construct a multi-layered mechanism of TSHR activation, enabling a subtle transition from normal physiology to pathological transformation.

Downstream of TSHR, the main signaling branches are Gαs and Gαq/11. Among these, Gαs activates adenylate cyclase (AC), producing cyclic adenosine monophosphate (cAMP) as a second messenger, which further activates protein kinase A (PKA). PKA releases its inhibitory subunit and phosphorylates downstream targets, such as the cAMP response element-binding protein (CREB) (27). Through CREB, cAMP regulates target genes in a differential manner, a process influenced by CRE motif variants, promoter context, cofactor recruitment, and chromatin state, with CRE position contributing to target−gene specificity within the broader promoter and chromatin context. Target genes are involved in key cellular processes such as metabolism, cell cycle and survival, transcription, growth factor signaling, and immune regulation (28–30). Supporting the relevance of this pathway, Ramirez-Moya and Santisteban demonstrated that TSH induces DICER1 expression through the cAMP/PKA/CREB axis in differentiated thyroid cancer cells, and that CREB expression correlates with DICER1 in human thyroid tumors, indicating that the TSH-cAMP-CREB axis is active in thyroid cancer and linked to tumor biology (31). Pizzoni et al. showed that TSH triggers nuclear cAMP synthesis leading to PKA activation and CREB phosphorylation, which is sufficient to drive thyroid cell proliferation (32). These genes participate in multiple physiological processes and play important roles not only in tumor initiation and progression, tumor cell invasiveness, and extracellular matrix remodeling but also serve as mediators of environmental stress responses, promoting tumorigenesis. In thyroid cancer, high TSH-induced CREB overactivation can upregulate the expression of genes with enhanced mitogenic effects, driving uncontrolled proliferation and invasion (27, 28). It is also noteworthy that invasive or metastatic behavior generally requires cooperating oncogenic events, such as TRβPV or other co-occurring mutations capable of activating other signaling cascades, to establish an invasive phenotype (27). Equally important, as Chu noted, TSHR expression appears to be downregulated in patients with more advanced thyroid cancer, particularly those with poorly differentiated cell types, suggesting the presence of a non-specific adaptation mechanism that promotes a shift toward a less TSH-dependent state during tumor progression (27). In the more severe and aggressive anaplastic thyroid cancer (ATC), other members of the CREB family (such as CREB3L1) have been shown to promote pro-tumorigenic programs in ATC cells through KPNA2-mediated nuclear translocation, activating extracellular matrix (ECM) remodeling, inducing cancer-associated fibroblast (CAF)-like traits, and enhancing invasion and metastasis (33). These findings highlight that, despite the loss of classical TSHR signaling in ATC, CREB3L1 can still function as a driver of malignancy. Additionally, some studies have found that CD133-positive cells in ATC cell lines exhibit stem cell-like characteristics, such as high proliferation, self-renewal, and the ability to form colonies *in vitro*. As a key regulatory factor, TSH can promote the generation and upregulation of CD133 cells by activating signaling pathways, enhancing the survival and function of cancer-initiating cells (34). It should be noted that ATC is biologically distinct from DTC. Therefore, findings from ATC (e.g., CREB3L1, CAF-like characteristics, CD133-positive cells) offer indirect support and should not be directly extrapolated to postoperative TSH suppression therapy in DTC.

At the same time, as discussed by Chu et al., Gαq can regulate the proliferation and survival of thyroid cells by activating the PLC-PKC-MAPK and PI3K-AKT pathways (27). However, in thyroid cancer, these signaling pathways are often hyperactivated due to driver gene mutations. Based on a 2017 review by Miguel et al., approximately 70% of thyroid cancers carry activating mutations in the MAPK pathway (such as BRAF and RAS point mutations and RET/PTC rearrangements), and mutations in different components of the PI3K pathway are more prevalent in poorly differentiated and anaplastic thyroid cancers (35). Research in 2023 suggested that the flux of the MAPK signaling pathway is a key factor determining the biological characteristics of thyroid cancer, influencing cellular differentiation status, invasiveness, and the composition of the tumor microenvironment (36). Furthermore, sustained activation of the PI3K-PKB/AKT pathway and genetic mutations in downstream effector molecules can also lead to high proliferation in various cancer cells, including those of thyroid cancer. Further studies have revealed that TSH plays a more complex role in this network (37). A 2014 study in BRAFV600E-mutant thyroid cancer found that TSH, together with DUSP6 (Dual Specific Phosphatase 6), activates Ras/AKT signaling and stabilizes c-Myc expression, thereby overcoming oncogene-induced senescence and driving thyroid carcinogenesis (38). AKT, as a key regulator of cellular function, exhibits abnormal activation that is not only associated with the survival, proliferation, and growth of aberrant cancer cells but also affects tumor metabolism, angiogenesis, and invasive capacity, promoting cancer progression and metastasis (39). Additionally, some studies have suggested that under long-term TSH stimulation, TSH can reduce p53 expression by upregulating the PI3K/AKT pathway, leading to the loss of oncogene-induced senescence (OIS). Thyroid cancer cells gradually overcome OIS, become TSH-independent, and progress to poorly differentiated carcinoma (40).

In summary, the clinical value of TSH suppression therapy must be interpreted within a context−dependent framework. In TSHR−positive differentiated thyroid cancers, TSH suppression can limit TSH−driven growth stimulation. However, the benefit is not uniform across all patients. As TSHR expression is frequently reduced or lost in advanced, poorly differentiated, or radioiodine−refractory tumors, such cancers are less likely to respond to TSH suppression and may even progress independently of TSH. Therefore, a tailored approach that considers TSHR expression status, tumor differentiation, and dynamic risk assessment is essential to avoid overtreatment and to maximize therapeutic benefit.

## 3 Factors influencing TSH suppression Therapy from an Individualized Perspective

In the postoperative management of differentiated thyroid cancer, TSH suppression therapy, which maintains serum TSH at low levels through exogenous thyroid hormone supplementation, is a key strategy for reducing the risk of tumor recurrence. However, significant inter-individual variability exists in the efficacy and safety of this therapy, leading to uncertainty in treatment outcomes and varying risks of adverse effects. Therefore, systematically analyzing the multidimensional factors influencing the response to TSH suppression therapy from an individualized perspective is essential for optimizing the long-term management of patients with differentiated thyroid cancer.

Several factors can hinder the achievement of target TSH levels. Preoperative TSH levels ≥2 mIU/L and the presence of Hashimoto’s thyroiditis are independent risk factors for hypothyroidism after hemithyroidectomy (41). Hashimoto’s thyroiditis, as a common form of autoimmune thyroid disease, exposes residual thyroid tissue to autoantibodies postoperatively, further intensifying the loss of negative feedback and high TSH secretion (42). Moreover, patients with a higher degree of lymphocytic infiltration have a higher probability of developing hypothyroidism after surgery (43). Inadequate levothyroxine dosing—often due to non−adherence or underestimation of requirements—can likewise prevent target attainment.

Dose requirements are influenced by age, sex, body size/BMI, and potential assay interference factors. Specifically, age-related reductions in tissue responsiveness to thyroid hormones, along with higher dosage requirements in women—particularly premenopausal women—and weight-based initial dose calculations, further complicate management strategies (44, 45). Multiple studies have indicated that in women, especially postmenopausal women, estrogen deficiency amplifies the impact of TSH suppression therapy on bone metabolism, resulting in accelerated bone turnover and insufficient bone formation, placing them at higher risk for osteoporosis and fractures during treatment (46–49). Elderly individuals are more susceptible to the adverse effects of TSH suppression, such as cardiovascular risk, alterations in bone metabolism, reduced cognitive scores, and impaired quality of life, whereas younger individuals face greater risks of disease progression and recurrence under long-term higher TSH stimulation (50). Therefore, according to the 2025 ATA guideline, age alone should not dictate suppression intensity. Decisions should be based on dynamic risk stratification, treatment response, and a patient-specific benefit-risk assessment. In addition to food, numerous drugs (such as ferrous sulfate, calcium carbonate, aluminum hydroxide, sucralfate, bile acid sequestrants, and raloxifene) can impair L-T4 absorption by altering the gastrointestinal environment or through direct binding, leading to “iatrogenic hypothyroidism” (i.e., persistently elevated TSH despite seemingly adequate dosing) (51). Furthermore, antibody interference is particularly complex and requires careful differentiation. Macro-TSH is a known cause of falsely elevated TSH levels with normal thyroid hormone levels. However, the dilution test is neither universally abnormal nor sufficiently specific to distinguish it from other interfering factors (such as biotin, anti-streptavidin antibodies, anti-ruthenium antibodies, and thyroid hormone autoantibodies) (52). Heterophilic antibodies more commonly cause false-elevated TSH results, although false-low results related to the assay method may also occur (52). Therefore, identifying these interferences is crucial for avoiding inappropriate dose adjustments and preventing iatrogenic hyperthyroidism or hypothyroidism.

Tumor pathological characteristics and disease-related factors, as core elements in assessing the risk of recurrence in differentiated thyroid cancer, directly influence individualized TSH suppression strategies based on potential benefits and risks. The latest 2025 ATA guidelines (formally published version) emphasize a more accurate assessment of recurrence risk in differentiated thyroid cancer based on tumor histopathological characteristics, the number of cervical lymph node metastases, the AJCC staging system, postoperative imaging findings, and serum Tg and TgAb results, with the degree and duration of TSH suppression individualized according to potential benefits and risks (8). However, as no new TSH suppression targets were proposed, the stratification from the 2015 ATA guidelines remains in use: TSH maintained at 0.5–2.0 mIU/mL for low-risk DTC, 0.1–0.5 mIU/mL for intermediate-risk DTC, and <0.1 mIU/mL for high-risk DTC (3). Particularly for patients with BRAFV600E gene mutations, which serve as a major driver of differentiated thyroid cancer, this mutation synergizes with TSH to promote tumorigenesis (38). More critically, long-term TSH stimulation can enable thyroid cancer cells to overcome oncogene-induced senescence, leading to the failure of p53-dependent senescence, gradually becoming TSH-independent, and progressing to poorly differentiated carcinoma (40). Therefore, it is essential and critical to develop individualized TSH suppression strategies, conduct regular monitoring and dynamic adjustments.

After establishing individualized TSH suppression targets based on patient and tumor characteristics, successful treatment implementation also highly depends on drug selection, patient adherence, and interference from various internal and external factors. These factors at the level of treatment implementation directly affect the stable achievement of serum TSH levels and represent an indispensable aspect of achieving individualized therapy. Oral levothyroxine (L-T4) effectively mimics physiological secretion, stably converts to T3 in the body, and serum T4 provides more effective feedback regulation of pituitary TSH secretion than T3, making L-T4 monotherapy the preferred option for the majority of patients (12). Additionally, the absorption of L-T4 is influenced by formulation, and gel or liquid formulations may provide faster and more stable absorption (53). More critically, strict administration guidelines—taking on an empty stomach in the morning, swallowing with water, and maintaining an interval of at least 30–60 minutes from breakfast and calcium/iron supplements—are essential to maximize bioavailability (54). However, this “ideal” regimen often conflicts with patients’ lifestyle habits, leading to unintentional non-adherence or malabsorption, which are the most common sources of interference in dose adjustment. Nevertheless, some studies have suggested that administering L-T4 at breakfast is safe and well-tolerated, serving as an alternative for patients with adherence difficulties (54). As this alternative approach is more likely to cause fluctuations in TSH levels, closer follow-up of such patients is necessary. At the same time, it is essential to develop a structured communication plan. Patients should be informed about the symptoms of both thyroid hormone excess (e.g., palpitations, tremors, weight loss, heat intolerance) and deficiency (e.g., fatigue, cold intolerance, weight gain). The importance of daily L-T4 medication adherence should be emphasized, and patients should be informed that biochemical markers (TSH, free T4) take 6–8 weeks to stabilize after any dose adjustment. Following a dose adjustment, TSH and free T4 should be re-evaluated once a steady state is reached. Symptoms of excessive suppression (palpitations, tremors, insomnia, weight loss, heat intolerance) must be routinely monitored. In the elderly, postmenopausal women, and individuals with a history of cardiovascular disease, close monitoring is required for complications such as atrial fibrillation, tachyarrhythmias, bone loss, and fractures. Follow-up intervals should be individually adjusted based on clinical status, trends in laboratory parameters, and imaging results. The relaxation or re-intensification of suppression therapy should also be linked to changes in recurrence status, treatment response, and toxicity risk.

**Figure 2:**
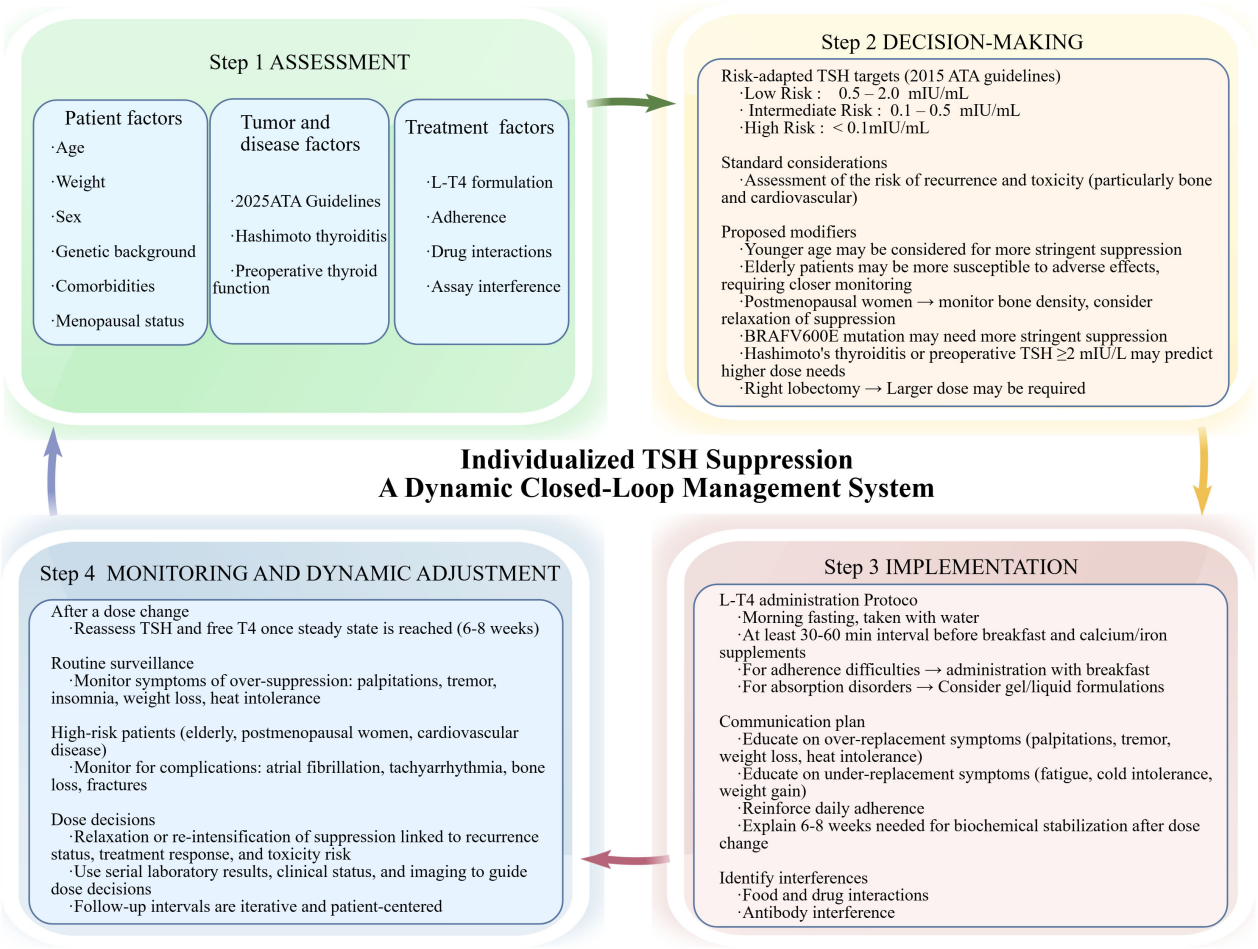
Individualized TSH suppression: a dynamic closed−loop management system: This system integrates patient assessment, risk stratification, treatment implementation, and dynamic adjustment. Suppression intensity is modified based on recurrence status, treatment response, and toxicity risks to balance recurrence prevention and quality of life.

In summary, the individualized management of TSH−suppressive therapy is a dynamic balancing process that integrates multidimensional information. This process begins with a detailed assessment of the patient’s intrinsic characteristics (age, sex, and genetic background) to predict treatment tolerability and dosage requirements. Subsequently, precision risk stratification based on tumor pathological characteristics determines the initial treatment intensity and goals. However, the ultimate success of personalized strategies depends on meticulous management during the treatment implementation phase. The intensity of suppression is adjusted in a timely manner based on changes in recurrence status, treatment response, and toxicity risks. By establishing a closed−loop management system of “assessment−decision−execution−monitoring,” the goal is to achieve an optimal balance between reducing the risk of recurrence and ensuring quality of life in the postoperative management of thyroid cancer (**Figure 2**).

## 4 The benefit boundaries of TSH suppression therapy

The benefits of TSH suppression therapy for differentiated thyroid cancer have a strong research foundation. As early as 1954, the first case report described a patient with lung metastases who received TSH suppression therapy after radioactive iodine treatment, showing clinical improvement, objective improvement in lung function, and clear resolution of pulmonary opacities on X-ray, providing early clinical evidence for this treatment strategy (55). Subsequently, a 10-year follow-up study by Mazzaferri et al. published in 1981, involving 576 patients with papillary thyroid cancer, further emphasized that for patients with primary tumors >1.5 cm in diameter or with invasion of the thyroid capsule, particularly in those over 40 years of age, lifelong suppression of endogenous TSH was suggested to be important (56). As evidence accumulated, the benefits of TSH suppression gradually exhibited significant patient heterogeneity and dose-effect characteristics. In 2002, Nayahmka et al. conducted a meta-analysis of existing studies with suboptimal designs (lacking randomization and appropriate controls) and found that patients receiving TSH suppression therapy had an overall reduced risk of major adverse events (disease progression, recurrence, death). However, the study also noted that the optimal level of TSH suppression remained controversial among patients with different clinical characteristics (57). This conclusion presaged the necessity of individualized management, and an increasing number of studies have sought to define the boundaries of this “optimal level.” A single-center observational study by Guido et al. published in 2007 suggested that the harmful effect of TSH on recurrence or death became significant when the median TSH exceeded 2 mU/L, and therefore recommended that TSH levels in low-risk patients be maintained in the lower normal range (10). A multicenter cohort study further found that during active surveillance, serum TSH levels were positively correlated with the progression of papillary thyroid microcarcinoma (PTMC), and this association was particularly pronounced in patients under 50 years of age (58). For younger patients, maintaining TSH at normal or mildly subnormal levels with levothyroxine may be considered an option to inhibit tumor progression (59). However, the above conclusions are not without controversy. A prospective cohort study with a relatively small sample size and insufficient follow-up duration observed PTMC by ultrasound and failed to find a significant correlation between TSH and tumor progression (60). A multicenter database analysis even suggested that even in intermediate- to high-risk DTC patients, the prognostic benefit of TSH suppression was quite limited (61). Another cohort study with a larger sample size and a higher proportion of recurrent cases observed no correlation between mean TSH levels and recurrence risk in either low-risk or intermediate- to high-risk groups (62). These conflicting findings suggest that the relationship between TSH and tumor behavior may be influenced by multiple factors such as study design, population selection, and follow-up duration, and is far from being as simple as a linear correlation.

A prospective study with a median follow-up of 54 months, while emphasizing the critical importance of TSH suppression therapy for high-risk patients, also noted that normalizing TSH levels during long-term treatment is equally important for elderly patients without disease recurrence and for those with comorbidities (63). For DTC patients with distant metastases, studies have shown that TSH levels >0.1 mU/L are associated with poor prognosis, but the clinical significance of TSH suppression to <0.1 mU/L may be subject to dual interpretation. On one hand, the adverse effects of supraphysiological doses of thyroid hormones on the heart and skeleton may offset the anti-cancer benefits. On the other hand, metastatic patients may present with elevated T4/T3 levels due to increased endogenous hormone production, in which case high thyroid hormone levels themselves become a marker of poor prognosis rather than a direct reflection of tumor pathological factors (64). This insight reminds us that when interpreting the association between TSH and prognosis, caution must be exercised to avoid conflating “marker” with “cause.” The series of studies by the National Thyroid Cancer Treatment Cooperative Registry provide a more in-depth perspective for understanding the stratified benefits of TSH suppression. An early multicenter data analysis published by David et al. in 1998 showed that the level of TSH suppression was associated with disease progression in high-risk patients, but this association lost statistical significance after incorporating radioactive iodine therapy into the multivariate model, and the data did not support the need for greater TSH suppression to prevent disease progression in low-risk patients (65). A prospective multi-institutional registry study published by Jonklaas et al. in 2006 further refined the understanding of low-risk patients, demonstrating that moderate TSH suppression after near-total thyroidectomy and radioactive iodine therapy improved overall survival in low-risk patients, whereas more aggressive TSH suppression was associated with improved overall survival only in high-risk patients, suggesting that such aggressive therapy appears unnecessary for low-risk patients (66). In 2015, the National Thyroid Cancer Treatment Cooperative Study Group analyzed long-term data from 1987 to 2012 and revealed the temporal characteristics of TSH suppression benefits, finding that sustained moderate suppression (defined as subnormal to normal TSH levels) was associated with improved overall survival and disease-free survival for at least three years after diagnosis, but this advantage was no longer significant after five years, and more aggressive therapy with undetectable serum TSH did not confer additional benefit at any disease stage (67). However, analyses of registry databases require cautious interpretation due to their inherent limitations. Cases reported to registries may be subject to selection bias, and the diversity of management practices across participating institutions is difficult to fully control. Among patients receiving TSH suppression therapy, nearly all high-risk patients had undergone total thyroidectomy and radioactive iodine therapy. While this high degree of consistency in treatment reflects clinical standardization, it also makes it difficult to demonstrate the independent benefits of the three treatment modalities through multivariate analysis, especially when their effects may converge through common biological pathways involving iodine uptake and TSH responsiveness. Furthermore, the patient population receiving TSH suppression therapy may have undergone more intensive overall monitoring and management, including more aggressive TSH suppression strategies and more frequent follow-up, and this “healthy patient effect” may further confound the true contributions of surgery and radioactive iodine therapy.

The above evidence reminds us that for high-risk patients, the combination of surgery, radioactive iodine therapy, and TSH suppression may be an important approach for achieving optimal prognosis. However, for low-risk patients, moderate suppression (such as maintaining TSH in the lower normal range) is sufficient, and there is no need to pursue undetectable TSH levels. More importantly, the benefits of TSH suppression exhibit clear population specificity (greater benefits in high-risk and younger patients), and a better approach is “dynamic adjustment”—timely adjusting the intensity of suppression according to changes in patient age, presence of recurrence, emergence of new comorbidities, and other factors during long-term follow-up. Future research needs to further integrate molecular markers and prospective data to advance TSH suppression therapy from “empirical stratification” toward “precise prediction.”

## 5 Long-term safety profile and monitoring protocol for TSH suppression therapy

Despite establishing the boundaries of benefit for TSH-suppressive therapy in specific patient populations, the long-term safety of this treatment must not be ignored. In particular, cardiovascular events such as atrial fibrillation, decreased bone density and the risk of fractures, as well as neuropsychiatric symptoms and impaired quality of life, are issues that must be addressed in clinical management. Therefore, accurately identifying high-risk individuals and implementing standardized monitoring and management are crucial for balancing efficacy and risk and ensuring patients’ long-term health.

Multiple studies have confirmed a clear association between TSH-suppressive therapy and an increased risk of cardiovascular events. An observational study by Hesselink et al. suggests an increased risk of cardiovascular and all-cause mortality in patients with DTC (68). An individual-level data analysis of six prospective cohorts involving 25,390 participants further indicated that the risk of heart failure events is particularly elevated when TSH levels are <0.10 mIU/L (69). In primary care populations, even among individuals with normal free thyroid hormone levels, reduced TSH carries risks: those with high-normal thyroid function have an approximately 10% increased risk of atrial fibrillation, while those reaching subclinical hyperthyroidism (complete TSH suppression) have an approximately 40% increased risk (70). A cross-sectional study involving 136 thyroid cancer patients similarly demonstrated that TSH-suppressive therapy is associated with a high prevalence of atrial fibrillation, particularly among elderly patients (71). Meta-analyses consistently confirm that TSH-suppressive therapy following thyroid cancer surgery increases the risk of atrial fibrillation (72). In addition to arrhythmias, TSH suppression has multifaceted effects on cardiac function and structure. Conventional echocardiographic studies have found that long-term suppression therapy can lead to tachycardia, an increase in atrial premature beats, an increase in left ventricular mass index, and compensatory enhancement of left ventricular systolic function (manifested as an increase in the fractional shortening (73). However, more sensitive studies using two-dimensional speckle tracking technology have shown that baseline circumferential and longitudinal myocardial strain in DTC patients undergoing TSH suppression therapy was already significantly lower than in healthy controls, suggesting that the myocardium’s intrinsic deformability may already be impaired under conditions of exogenous subclinical hyperthyroidism (74). These findings collectively indicate that the effects of TSH suppression on the heart are complex: improved overall cardiac output does not equate to the safety of the myocardium itself, and impairment of myocardial deformability may occur even at the subclinical stage. In addition to changes in cardiac structure and function at rest, a study of 10 patients who had been receiving suppressive doses of L-T4 for a long period (5–9 years) and had developed exertional dyspnea and symptoms of adrenergic hyperactivity showed that long-term suppressive L-T4 therapy may lead to significant impairment of cardiac reserve and exercise capacity (75). The current evidence is primarily based on retrospective studies, prospective data are limited, and there is heterogeneity among different studies. Furthermore, there is a lack of prospective controlled studies examining the relationship between different degrees of TSH suppression and cardiovascular outcomes. Therefore, further prospective studies are needed to clarify the long-term safety of different suppression levels. Meanwhile, as discussed by Frederik et al., although TSH suppression therapy increases the risk of atrial fibrillation, cardiovascular mortality in DTC patients has not risen accordingly, which may be partially attributed to the “healthy patient effect” (regular follow-up leading to earlier treatment of cardiovascular disease) (76). It is essential to thoroughly assess individual cardiovascular risk and closely monitor elderly patients and those with pre-existing cardiovascular conditions. Recommended monitoring includes electrocardiography and heart rate monitoring. Echocardiography should also be performed to assess left ventricular mass, myocardial strain, and diastolic function.

Multiple studies consistently indicate that TSH suppression therapy is clearly associated with reduced bone density and an increased risk of osteoporosis, and that these effects exhibit significant population variability and time-dependence. A meta-analysis suggests that chronic TSH suppression may be associated with an increased risk of osteoporotic fractures in patients with thyroid cancer, but given the limited sample sizes of existing studies, conclusions should be interpreted with caution (77). Cohort studies further indicate that in low- and intermediate-risk DTC patients, TSH suppression significantly increases the risk of postoperative osteoporosis and does not improve recurrence rates in these patients, though the osteoporosis analysis in this study was conducted only in women, so its implications for men cannot be directly inferred (78). Similarly, a meta-analysis by Heemstra et al. found that the risk of osteoporosis associated with subclinical hyperthyroidism is primarily observed in postmenopausal women (47). A study using quantitative computed tomography (QCT) found that TSH-suppressive therapy led to increased bone resorption and impaired trabecular and cortical bone characteristics only in postmenopausal women, with no adverse effects observed in premenopausal women, further confirming that postmenopausal women are at high risk for skeletal adverse reactions (49). A prospective controlled trial showed that TSH-suppressive therapy (mean TSH 0.07 mU/L) can lead to a significant decline in lumbar bone mineral density in female DTC patients starting one year after surgery, with women over 50 being more severely affected (79). For men, the evidence is relatively limited and the conclusions are inconsistent. A predominantly male case-control study showed that men with thyroid cancer had a 33% higher risk of osteoporosis compared to controls, but no difference in fracture risk (80). More specifically, a study involving female DTC patients with varying estrogen statuses suggested that monitoring bone metabolism markers and preventing bone damage is essential for patients requiring higher doses of levothyroxine or those with estrogen deficiency (48). Meanwhile, other studies have suggested that low TSH levels are associated with reduced grip strength, particularly among men under 70 years of age, indicating that impaired muscle function may be a potential adverse effect of TSH-suppressive therapy (81). Regarding dose-response and disease duration, a study in older adults aged 70 and older showed that levothyroxine therapy was associated with a dose-dependent increase in fracture risk, further supporting the notion that TSH-suppressive therapy may have similar adverse effects on bone (82). A 1-year prospective study found that TSH-suppressive therapy accelerates bone loss primarily in postmenopausal women during the early postoperative period (within 1 year), with more pronounced bone loss observed in patients not receiving calcium/vitamin D supplements (83). In contrast, a retrospective study of postmenopausal women with DTC suggested that long-term TSH suppression (≥5 years) may increase bone fragility by altering trabecular bone structure (84). Based on the current evidence, the skeletal risks associated with TSH-suppressive therapy are primarily concentrated in postmenopausal women and those receiving high-dose therapy. Currently, there is a lack of large-scale, prospective, randomized controlled trials to clearly define the optimal TSH level that minimizes the adverse effects of iatrogenic hyperthyroidism while preserving the beneficial effects on recurrence in low- and intermediate-risk DTC patients. In clinical practice, high-risk individuals should undergo routine bone density monitoring, receive calcium and vitamin D supplementation as appropriate, and avoid unnecessary long-term deep suppression.

The impact of long-term TSH-suppressive therapy on neuropsychiatric function and quality of life remains somewhat heterogeneous in the current evidence, but most studies suggest adverse risks. Regarding cognitive function, a large-scale cohort study involving 65,931 older adults aged 65 and older found that exogenous thyrotoxicosis (such as TSH-suppressive therapy) may increase the risk of cognitive impairment, with a dose-response trend, suggesting that excessive thyroid hormone therapy may impair cognitive function in the elderly (85). A case-control study also demonstrated that patients with diffuse thyroid carcinoma (DTC) receiving chronic TSH-suppressive therapy performed significantly worse than the control group in executive function, psychomotor speed, and attention, further supporting the adverse effects of TSH suppression on neuropsychological function (86). However, some cross-sectional studies have found that female patients receiving TSH-suppressive therapy experienced declines in health status and mood but no impairment in cognitive function, suggesting that these emotional changes may be related to psychological factors rather than exogenous subclinical thyrotoxicosis itself (87). Further, a small number of studies with limited sample sizes found no evidence of cognitive impairment and even suggested that exogenous levothyroxine may have a potentially beneficial effect on cognitive function in patients lacking endogenous thyroid hormones (88). Regarding mood and quality of life, multiple studies consistently show that TSH-suppressive therapy is associated with psychological distress and a decline in quality of life. One cross-sectional study found that DTC patients receiving long-term TSH-suppressive therapy scored significantly lower than healthy controls across multiple quality-of-life dimensions, suggesting that supraphysiological doses of levothyroxine may impair psychological functioning and quality of life (89). Another study reported that DTC patients undergoing TSH-suppressive therapy had significantly lower health-related quality of life than the general population, with an anxiety prevalence as high as 44% (90). Furthermore, a longitudinal study showed that thyroid cancer survivors continued to experience significant psychological distress, anxiety, and depression many years after treatment ended, with approximately half of the patients having unmet psychological needs, highlighting the importance of medical and psychological monitoring (91). It is worth noting that research findings regarding quality of life are not entirely consistent. Some randomized controlled trials have found that long-term TSH-suppressive therapy does not significantly impair quality of life, nor does restoring normal thyroid function markedly improve it (92). Prospective population-based studies have also indicated that TSH suppression itself has no significant negative impact on quality of life, and that fear of recurrence may be the primary influencing factor (93). Of particular interest, a cross-sectional study showed that DTC patients receiving TSH suppression therapy scored significantly lower than healthy controls in terms of physical function, vitality, and mental health, while a 12-week exercise intervention significantly improved these measures (94). This finding suggests that exercise may be an effective non-pharmacological intervention strategy to mitigate the negative psychological effects associated with TSH suppression therapy. In summary, TSH suppression therapy is associated with cognitive impairment, anxiety, depression, and reduced quality of life, though the evidence exhibits some heterogeneity. In clinical practice, clinicians should be vigilant regarding the potential contribution of TSH suppression in patients reporting changes in mood, energy, or cognition, while encouraging regular exercise, managing psychological factors such as fear of recurrence, and adjusting treatment intensity on an individualized basis. Future high-quality, large-scale prospective studies are needed to clarify the long-term effects of different degrees of TSH suppression on neuropsychiatric function.

Overall, long-term TSH-suppressive therapy is associated with a range of adverse events involving the cardiovascular system (atrial fibrillation, impaired myocardial strain, and reduced cardiac reserve), the skeletal system (bone loss, osteoporosis, and fractures), and neuropsychiatric function (cognitive impairment, anxiety, depression, and reduced quality of life). High-risk groups include postmenopausal women, elderly patients, those receiving high-dose levothyroxine therapy, and individuals with pre-existing cardiovascular disease or osteoporosis. Practical monitoring and mitigation strategies should include baseline and periodic electrocardiograms, heart rate monitoring, echocardiography, bone density measurements, assessments of mood and cognitive symptoms, and individualized dose adjustments based on dynamic risk stratification. Concurrently, calcium and vitamin D supplementation, as well as fall prevention measures, are recommended. Importantly, active attention should be paid to patients’ mental health (particularly anxiety and fear of recurrence), and regular exercise should be encouraged as a non-pharmacological intervention. Future large-scale, prospective randomized controlled trials are needed to determine the minimum effective TSH suppression level for each risk category, compare long-term outcomes among patients with different TSH target ranges, and establish evidence-based thresholds that minimize toxicity without compromising recurrence-free survival.

## 6 Conclusion and perspectives

As a core strategy in the postoperative management of differentiated thyroid cancer, the clinical benefits of TSH suppression therapy have been well established. However, with the deepening of research, the complexity of this therapeutic approach has become increasingly evident. This article systematically reviews the mechanisms by which TSH contributes to the progression of thyroid cancer and comprehensively analyzes the key variables influencing the individualized implementation of TSH suppression therapy from three dimensions: patient factors, tumor pathological characteristics, and the treatment implementation process. Evidence indicates that the benefits of TSH suppression exhibit clear population specificity and temporal characteristics: high-risk patients and younger populations derive significant benefit, whereas low-risk patients do not require excessive suppression. Moreover, the first five years post-surgery represent the optimal window for intervention, with limited benefit from continued deep suppression beyond five years (67). These findings collectively suggest that shifting TSH suppression therapy from a “one-size-fits-all” empirical model toward a dynamic closed-loop management system of “assessment-decision-execution-monitoring” may represent the direction for future clinical practice.

It is worth noting that numerous studies have systematically and thoroughly explored the risks associated with TSH suppression therapy. The exogenous subclinical hyperthyroidism induced by TSH suppression has the most clearly established effects on the cardiovascular system, potentially leading to increased heart rate, increased left ventricular mass, impaired myocardial strain and diastolic function, as well as reduced arterial elasticity, cardiac reserve, and exercise capacity. These effects are more closely related to the duration of TSH suppression than to circulating thyroid hormone levels alone (95). Regarding the skeletal system, long-term TSH suppression therapy can lead to decreased bone density, osteoporosis, and an increased risk of fractures, with postmenopausal women being particularly high-risk. Therefore, close monitoring of bone health and implementation of appropriate preventive measures are necessary during treatment (77, 79). Additionally, for men under 70 years of age, the adverse effects of low TSH concentrations on muscle function also warrant attention (81). On the other hand, long-term TSH suppression therapy may impair neuropsychological function and induce symptoms of hyperthyroidism, leading to declines in patients’ emotional and cognitive status (86, 87). However, the effects on cognitive function remain controversial (87, 88). Furthermore, overly stringent TSH suppression targets may lead to unnecessary treatment and additional monitoring, increasing patient burden and healthcare costs (96). These risks are closely related to the intensity and duration of suppression and must be considered when formulating individualized strategies.

Achieving precise suppression represents the core direction for future research. In clinical practice, the timing of postoperative TSH monitoring warrants re-evaluation. Some studies suggest that TSH measurement at one month post-surgery may be too early to accurately reflect the stable level at three months (97). Whether low-risk patients undergoing unilateral lobectomy require TSH maintenance below 2 mIU/L remains unsupported by high-level evidence (98). Conducting large-scale prospective randomized controlled trials is an urgent need for future research, although such studies face practical challenges including large sample sizes, long follow-up periods, and high costs. Refinement of risk stratification systems represents another important direction. The weighting of pathological features such as multifocal disease, the extent and location of extrathyroidal extension, and positive margins requires further clarification, while the integration of emerging molecular markers is expected to significantly improve the accuracy of risk prediction (99). Moreover, the intensity of TSH suppression therapy is not static. The optimal timing for relaxation of suppression and the accumulation of suppression-related risks are directly related to the dynamic adjustment of treatment targets. Additionally, the significant gap between guideline development and implementation highlights the critical importance of effective guideline dissemination and clinician education (100).

Ultimately, treatment decisions should return to a patient-centered essence: avoiding unnecessary TSH suppression for patients with very low recurrence risk, and for those requiring long-term therapy, establishing a dynamic assessment mechanism to adjust suppression intensity appropriately according to recurrence status, advancing age, and new-onset comorbidities. The ultimate goal of individualized TSH suppression therapy is to maximize patients’ long-term quality of life while effectively preventing tumor recurrence.
